# Effects of a Trans-Galactooligosaccharide on Minerals Content of Common Carp (*Cyprinus carpio* L.) Tissues

**DOI:** 10.1007/s12011-021-02600-w

**Published:** 2021-01-26

**Authors:** Ewa Ziółkowska, Joanna Bogucka, Jan Mazurkiewicz, Mateusz Rawski, Szymon Różański, Magdalena Stanek

**Affiliations:** 1 Department of Animal Physiology, Physiotherapy and Nutrition, Faculty of Animal Breeding and Biology, UTP University of Science and Technology, Mazowiecka 28, 85-004 Bydgoszcz, Poland; 2 Division of Inland Fisheries and Aquaculture, Faculty of Veterinary Medicine and Animal Science, Poznań University of Life Sciences, Wojska Polskiego 71C, 60-625 Poznań, Poland; 3 Laboratory of Feed and Raw Animal Materials, Faculty of Animal Breeding and Biology, UTP University of Science and Technology, Mazowiecka 28, 85-004 Bydgoszcz, Poland

**Keywords:** Prebiotic, Carp, Meat, Gills, Skeleton, Minerals

## Abstract

Common carp (*Cyprinus carpio* L.) is a dominant fish species in aquaculture, and as it is a stomachless species, absorption and digestion of nutrients take place in the intestine. The aim of the study was to evaluate the effects of a prebiotic on the content of selected minerals found in the meat, gills, and skeleton of common carp. The research applied trans-galactooligosaccharide (GOS) prebiotic produced by enzymatic transgalactosylation of milk lactose by whole cells of *Bifidobacterium bifidum*. The following diets have been applied: control diet without feed additives (C), diet 2 (B1) with 1% of GOS, and diet 3 (B2) with 2% of GOS. In the freeze-dried samples, concentrations of the analyzed metals were determined using atomic absorption spectroscopy (AAS). The content of phosphorus was determined using colorimetric method. The analyses confirmed that the highest level of Mg was detected in the skeleton of fish fed with 1% GOS (2.51 g kg^−1^) and was significantly higher compared the control treatment (2.11 g kg^−1^) (*P* < 0.05). Zn content in fish meat fed with 1% GOS (35.41 mg kg^−1^) was significantly higher (*P* < 0.05) than in the control group (24.59 mg kg^−1^). The tissue that accumulated the greatest amount of Zn was the gills. GOS had a positive effect on Fe accumulation in the meat, gills, and skeleton. It has been concluded that supplementation of feed with 2% GOS significantly influenced the positive correlations between Mg and P in the meat and skeleton, Fe–Ca correlation in gills, and Fe–Zn correlation in the skeleton.

## Introduction

Fish is one of the most valuable food sources of minerals, the concentration of which is influenced by many endogenous and exogenous factors. There are many factors present in the feed that can inhibit the absorption of minerals, e.g., phytates, fibers, and heavy metals [1–3]. Moreover, the degree of mineral absorption is influenced by the physicochemical properties of water, such as hardness, salinity, and pH [3, 4]. Changes in the dietary composition and mineral concentration of rearing water could have an impact on the mineral balance in fish tissues and proper physiological state of animals. One of the most serious health and economic problems in the world of marine and freshwater aquaculture is the fish body deformation [5, 6]. Initially, deformations of the food-based skeleton were explained by the vitamin C deficiency in diet, yet currently the main cause of these deformations has been attributed to the deficiency of minerals [6]. A research has confirmed that the presence and proper balance between Ca, P, Mg and Zn are necessary for the correct mineralization of the fish skeleton [3, 6, 7]. Moreover, since excessive Fe intake inhibits Zn absorption (antagonistic effect), analyzing Fe levels should also be considered.

Common carp (*Cyprinus carpio* L.) has been a dominant fish species in the world aquaculture production [8] and was the first species introduced into Polish aquaculture. The first records on farming date back to the twelfth century, and after the year 1550, common carp accounted for about 75–80% of all fish farmed in ponds. Poland and the Czech Republic are the largest producers of common carp in the European Union, and the annual production of consumable common carp in Poland ranges between 15 and 23,000 t [9, 10]. Common carp is an omnivorous species with low nutritional requirements, both in terms of the feed composition and its production technology. In addition, common carp farmed in ponds largely relies on a natural diet which usually consists of the biomass produced by the pond. Carp does not have functional stomach, and digestion takes place in the intestine hence from digestibility food, and assimilation is limited. Due to the fact that the cyprinids do not have acid-secreting stomachs, the mineral absorption from organic compounds (in particular phosphorus) may be reduced [11, 12].

In recent years, the influence of various factors on the degree of fish mineralization has been studied such as different starter diets or water temperature [6, 13–15]. To our knowledge, there is little data available on the effect of prebiotics on the accumulation of minerals in different tissues of common carp. As confirmed by research prebiotics (e.g., fructooligosaccharides (FOS), transgalactooligosaccharides (t-GOS), inulin, and mannanooligosaccharides (MOS)) have a positive effect on the gut microbiota composition. They inhibit the growth of pathogenic gut microbial flora while stimulating the growth of microorganisms beneficial for the animal host. Beneficial gut bacteria can improve the body’s natural defenses, synthesize vitamins, and bind toxins and heavy metals. In aquaculture, prebiotics are increasingly used due to their positive effect on growth stimulation, use of feed, gut microflora, gut morphology, immune system, and disease resistance [8, 16–23]. These bioactive substances play an important role in regulating mineral metabolism, mineral bioavailability, and bone health [23–27]. Moreover, prebiotics affect the production of short-chain fatty acids, lowering the intestinal pH, regulating the factors responsible for the transport of divalent metals, thanks to which they improve the absorption of metals and skeletal health [28–32]. Some authors confirmed, that prebiotics and their products of fermentation by intestinal microflora have an enhancing effect on Fe and Zn absorption [23, 24, 33, 34]. Therefore, it definitely seemed valid to analyze the degree of absorption of minerals by carp family member (representatives of the *Cyprinidae* family) under prebiotic supplementation.

The prebiotic used in this experiment, under the trade name Bi^2^tos, is manufactured by Clasado (Biosciences Ltd., Jersey, UK) by enzymatic transgalactosylation of milk lactose by whole cells of *Bifidobacterium bifidum* 41171. For this reason, Bi^2^tos specifically promotes growth of *Bifidobacterium* spp. [35]. Our previous research revealed that the supplementation of feed with 1% and 2% Bi^2^tos significantly enhanced the development of the intestine, increased the height and width of the villi, and increased their surface area [36], which may contribute to increased absorption of nutrients from the gut.

The aim of the present study was to analyze the effects of dietary supplementation of a trans-galactooligosaccharide (GOS) on the content of selected minerals in the meat, gills, and skeleton of common carp and on the correlations between minerals analyzed.

## Material and Methods

Studies on live animals were carried out in strict accordance with the recommendations of the National Ethics Commission (Warsaw, Poland). All members of the research staff were trained in animal care, handling, and euthanasia. Fish health and welfare and the environmental conditions in the experimental tanks were checked twice daily by visual observation of animal behavior and by checking water quality parameters, such as oxygen saturation, temperature, and water flow. After sedation, the animals were decapitated according to the American Veterinary Medical Association Guidelines for the Euthanasia of Animals [37]. According to Polish law and an EU directive (no 2010/63/EU) [38], the experiments conducted in this study did not require approval from the Local Ethical Committee for Experiments on Animals in Poznań.

### Experimental Diets

The experimental diets were calculated as isonitrogenous (35.1% crude protein) and isoenergetic (18.5 MJ kg^−1^) with less than 4% of crude fiber and were formulated according to common carp nutritional requirements [39–41]. Three experimental diets were used: control diet 1 (C) without feed additives, diet 2 with 1% of GOS (B1), and diet 3 (B2) with 2% of GOS (Table 1).

**Table 1:** Dietary formulation and proximate composition of feed

Ingredient	Composition (%)		
	C	B1	B2
Fish meal^1^	12.3	12.3	12.3
Blood meal^2^	10.0	10.0	10.0
DDGS ^3^	11.0	11.0	11.0
Soybean meal^4^	15.0	15.0	15.0
Rapeseed meal^5^	10.0	10.0	10.0
Wheat meal	32.8	31.8	30.8
Fish oil^6^	4.6	4.6	4.6
Soybean lecithin^7^	1.0	1.0	1.0
Vitamin-mineral premix^8^	1.5	1.5	1.5
Vitamin premix^9^	0.1	0.1	0.1
Choline chloride	0.2	0.2	0.2
Fodder chalk	1.5	1.5	1.5
Prebiotic^10^	0.0	1.0	2.0
Proximate composition (% dry matter)			
Crude protein	35.06		
Crude lipid	9.08		
Crude fiber	3.93		
Total phosphorus	0.83		
Calcium	1.36		
Ash	7.17		
Gross energy (MJ·kg^−1^)	18.51		
Essential amino acids	g/100 g of crude protein		
Arginine	4.53		
Histidine	2.8		
Lysine	3.5		
Tryptophan	1.04		
Phenylalanine + tyrosine	4.96		
Methionine + cysteine	1.75		
Threonine	3.13		
Leucine	6.72		
Isoleucine	3.9		
Valine	4.97		

^1^Danish fishmeal, Type F, 72% total protein, 12% fat, FF Ska-gen, Denmark

^2^AP 301 P, 92% total protein, APC (GB) Ltd., Ings Road, Doncaster, UK

^3^Dried distillers grains with solubles, > 45% total protein, < 6% ash

^4^Toasted, 46–47% total protein, 1% fat

^5^33% total protein, 2% fat

^6^Agro-fish, Kartoszyno, Poland

^7^BergaPure, deoiled lecithin, 97% pure lecithin, Berg + SchmidtGmbH & Co. KG, Hamburg, Germany

^8^Polfamix W, BASF Polska Ltd. Kutno, Poland—1 kg contains vitamin A 1000000 IU, vitamin D3 200,000 IU, vitamin E 1.5 g, vitamin K 0.2 g, vitamin B1 0.05 g, vitamin B2 0.4 g, vitamin B12 0.001 g, nicotinic acid 2.5 g, Dcalcium pantothenate 1.0 g, choline chloride 7.5 g, folic acid 0.1 g, methionine 150.0 g, lysine 150.0 g, Fe 2.5 g, Mn 6.5 g, Cu 0.8 g, Co 0.04 g, Zn 4.0 g, J 0.008 g, carrier up to 1000.0 g

^9^Vitazol AD3E, BIOWET Drwalew, Poland—1 kg contains vitamin A 50000 IU, vitamin D3 5000 IU, vitamin E 30.0 mg, vitamin C 100.0 mg

^10^Bitos® trans-galactooligosaccharide (GOS), Clasado Ltd.; dry powder containing a mixture (wt: wt) of the following oligosaccharides: 45% lactose, 9.9% disaccharides [Gal–(β1–3)–Glc; Gal–(β1–3)–Gal; Gal–(β1–6)–Gal; Gal–(α1–6)–Gal], 23.1% trisaccharides [Gal–(β1–6)–Gal–(β1–4)–Glc; Gal–(β1–3)–Gal–(β1–4)–Glc], 11.55% tetrasaccharides [Gal–(β1–6)–Gal–(β1–6)–Gal–(β1–4)–Glc], and 10.45% pentasaccharides [Gal–(β1–6)–Gal–(β1–6)–Gal–(β1–6)–Gal–(β1–4)–Glc]

The experimental diets were prepared according to the following procedures below:

1. Preparation of components of the diets: individual components weighed out; ground in a percussion mill until very fine (mesh size 1 mm).
2. Preparation of the premix: vitamin and mineral components, soybean lecithin, choline chloride, chalk, and prebiotic were added to the carrier (soybean meal); mixed for 5 min in a cubic mixer.
3. Preparation of the diets: all ingredients and the premix mixed in a drum mixer for 5 min.
4. Conditioning the diets: hot water added; mixed in blade mixer for 5 min.
5. Extrusion: Metalchem S-60 single screw warm extruder (Gliwice, Poland), the extrusion conditions were as follows: a 90 °C cylinder temperature in the zone of increasing pressure, a 100 °C cylinder temperature in the zone of high pressure, a 110 °C head temperature, a 52-rpm speed screw, and a 6-mm nozzle diameter.
6. Drying: on mesh under a stream of heated air.
7. Sifting: the dust fraction sifted off in a percussion sifter.
8. Oiling: fish oil heated to 50 °C in quantities of 4.6% was used to coat extruded diet in a pelletizing drum.
9. Final sifting: the dust fraction sifted off in a percussion sifter.

Prepared feeds have been packed in foil bags and stored minus 18 °C until use.

### Fish Culture

The 60-day growth trial was carried out in the Experimental Station for Feed Production Technology and Aquaculture in Muchocin (Poland). Three hundred one-year-old common carp (mean body weight 180 g) were used. The fish were randomly stocked into 12 concrete ponds (40 m^3^), at a density of 25 fish per pond according to Horváth et al. [42]. The experiment was carried out in four replications (four ponds per treatment). Each pond was equipped with an automatic band feeder allowing for the continuous supply of feed during 12 h per day. The calculated daily feed dose for each pond was given every day at 9.00 a.m., its consumption was controlled visually twice a day, and rate was corrected if needed. The daily feed dose was restricted to assure that all feed supplied was consumed. The feeding rate was calculated in consideration of fish biomass in each pond which was corrected every 10 days on the basis of control bulk weighing of all fish; measurements of the current average daily water temperature and feed consumption from previous day were used for the additional correction according to Miyatake’s [43] recommendations, which resulted in feeding rate ranging from 1.8 to 3.3% of fish biomass. A constant flow of water in the experimental system was ensured by an open flow system with a mechanical pre-filtration chamber providing total exchange of water capacity in each pond every 12 h. During the experimental period, control of water physio-chemical parameters was carried out with the use of microcomputer oximeter Elmetron CO-315. Average daily water temperature and pH were studied which ranged from 17.7 °C to 22.7 °C and 7.2 to 7.6, respectively. Dissolved oxygen was kept above 3.5 mg O2/L, and hypoxia conditions were not observed in the experiment (details are described in Ziółkowska et al. [36]).

During the experiment, fish were anesthetized by immersion in 130 mg/L tricaine methanesulfonate (MS–222, Sigma Aldrich) for weighing at 10-day intervals for feed rate control. Body weight gain (BWG), feed intake (FI), feed conversion ratio (FCR), specific growth rate (SGR), protein efficiency ratio (PER), and percentage weight gain (PWG) were calculated (details are described in Ziółkowska et al. [36]).

### Sample Preparation

At the end of the experiment, four fish per pond were euthanized by immersion in 500 mg L^−1^ of MS–222 [44] for tissue sampling for metals analysis. The number of individuals subjected to analyses was based on earlier studies performed by Hoffman et al. [45] and Józefiak et al. [46] to provide a necessary sample size for laboratory and statistical analysis, and to avoid unnecessary animal sacrificing (according to 4R policy).

The meat samples for analyses were taken from the large side muscle of fish body above the lateral line, the gills that was branchial arch with filaments, and the skeleton that was a spine with ribs. The meat, gills, and skeleton were crumbled and freeze-dried in Lyovac GT2 freeze-drier by Finn-Aqua (Finland) (parameters: temperature − 40 °C, pressure 6·10^−2^ mbar, duration at least 48 h).

### Minerals Analyses

Metal concentrations were determined in freeze-dried samples after *aqua regia* digestion (ISO 11466:1995) using atomic absorption spectroscopy (AAS) with a SOLAR S4 spectrophotometer. Phosphorus content was analyzed with colorimetric method (ISO 13730:1996), by spectrophotometer Lambda 25, Perkin-Elmer (at wavelength 430 nm). The concentrations of the metals were calculated from linear calibration plots obtained from measurements of the working standard solutions. Certified AAS Merck standard solutions were used for the calibration of the standard curves, and validation was conducted on Certified Reference Material Fish Muscle ERM®-BB422 and Certified Reference Material Aquatic Plant BCR®-670. All determinations were made in triplicate, and the data for samples of the meat were corrected to oven-dry (105 °C) moisture content. Tissue concentrations of the metals were given in mg kg^−1^ dry weight (mg kg^−1^ d.w.) for Zn and Fe and g kg^−1^ dry weight (g kg^−1^ d.w.) for Mg, Ca, and P. Minerals analyses were conducted at UTP University of Science and Technology in Bydgoszcz (Poland).

### Statistical Analyses

Statistical calculations were made using Statistica 13.0 software (StatSoft 13.0). The arithmetic mean (x) and standard deviation (SD) were calculated. Four fish per pond (*n* = 16; 16 fish for each treatment) were collected for minerals analyses. Significant differences between the groups were tested with one-way analysis of variance (ANOVA), and Tukey’s test was used for multiple comparisons. The normality of the data was tested using the Shapiro-Wilk’s test and the homogeneity of variance was verified by means of the Levene’s test. The level of significance was determined at *P* ≤ 0.05. Interrelationships between analyzed minerals in the individual tissues were determined based on the Pearson’s correlation coefficients.

## Results

### Concentrations of Minerals

**Fig. 1:**
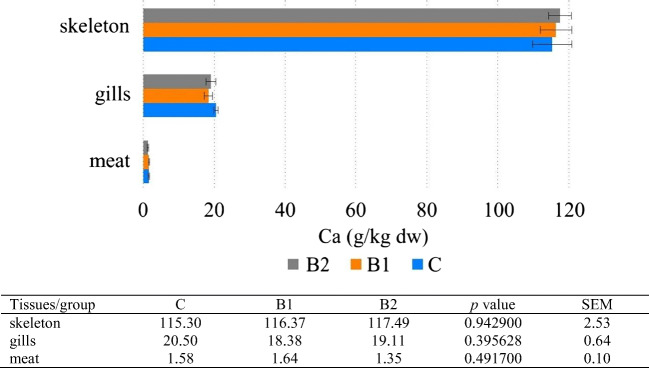
Effect of GOS supplementation on Ca concentration (g kg^−1^ d.w.) in tissues of common carp (*Cyprinus carpio* L.)

**Fig. 2:**
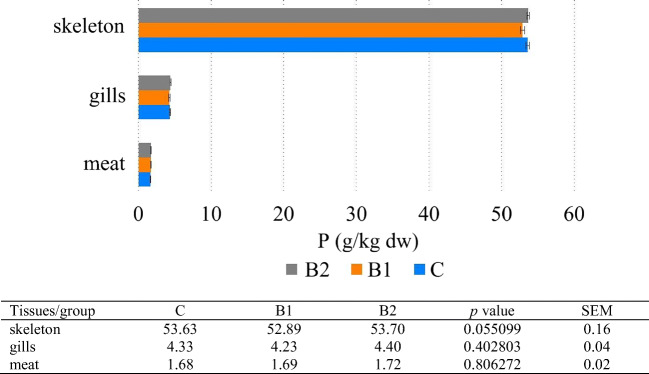
Effect of GOS supplementation on P concentration (g kg^−1^ d.w.) in tissues of common carp (*Cyprinus carpio* L.)

**Fig. 3:**
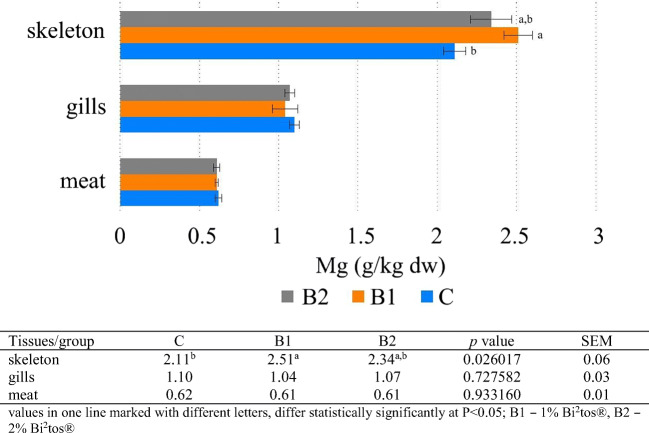
Effect of GOS supplementation on Mg concentration (g kg^−1^ d.w.) in tissues of common carp (*Cyprinus carpio* L.)

**Fig. 4:**
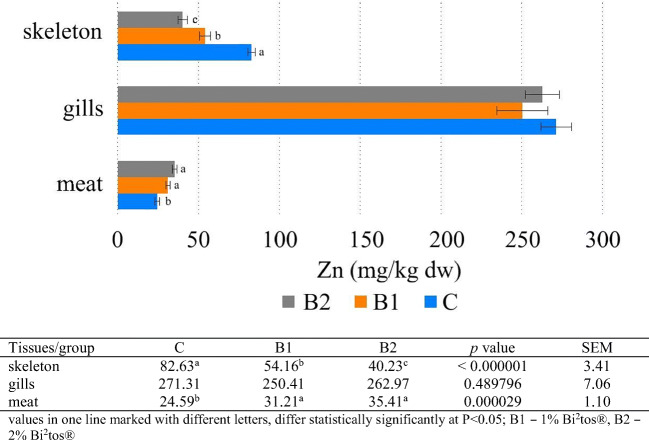
Effect of GOS supplementation on Zn concentration (mg kg^−1^ d.w.) in tissues of common carp (*Cyprinus carpio* L.)

**Fig. 5:**
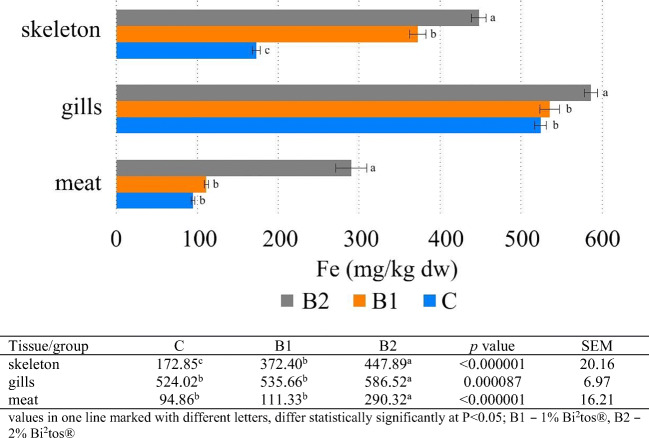
Effect of GOS supplementation on Fe concentration (mg kg^−1^ d.w.) in tissues of common carp (*Cyprinus carpio* L.)

The results of the present study showed that Ca, Mg, and P concentration increased in tissues in the following order: meat < gills < skeleton. Zn and Fe concentration increased in the following order: meat < skeleton < gills (Figs. 1, 2, 3, 4, and 5). Analyses confirmed no statistically significant differences in Ca and P content between fish fed 1 and 2% GOS compared control treatment (0% GOS) for each tissue (Figs. 1 and 2). Ca/P ratio in the meat was 0.83, in the gills 4.65, and in the skeleton 2.13. The analyses confirmed that the value of this coefficient in the skeleton was significantly higher in B1 (2.25) and B2 (2.22) groups compared to the control (1.91). The highest level of Mg was detected in the skeleton of fish fed 1% GOS (2.51 g kg^−1^ d.w.), and was significantly higher compared control treatment (0% GOS) (2.11 g kg^−1^ d.w.), but this result was similar to the value determined for fish fed 1% GOS (2.34 g kg^−1^). There were no statistically significant differences in Mg content between the experimental groups, both in the case of the meat and the gills (Fig. 3). The results of the present study showed that Zn contents in fish fed 1% GOS (31.21 mg kg^−1^ d.w.) and 2% GOS (35.41 mg kg^−1^ d.w.) were significantly higher than control group (24.59 mg kg^−1^) (Fig. 4). Furthermore, it was found that enhancing feed GOS significantly affect the decrease in the Zn concentration in the skeleton. As our analyses of carp indicated, GOS addition caused statistically significant differences in Fe level between the experimental groups within all tissues (Fig. 5). Higher level of Fe was in the meat of fish fed 2% GOS (290.32 mg kg^−1^ d.w.) in comparison with control group (94.86 mg kg^−1^ d.w.) and fish fed 1% GOS (111.33 mg kg^−1^ d.w.). The concentration of this metal in the gills was in the range from 524.02 mg kg^−1^ d.w. (C group) to 586.52 mg kg^−1^ d.w. (B2 group), and these values differed significantly. Fe content in the skeleton differed significantly between the C group (172.85 mg kg^−1^ d.w.), B1 group (372.4 mg kg^−1^ d.w.), and B2 group (447.89 mg kg^−1^ d.w.).

### Correlations Between Minerals

Statistically significant correlation coefficients between Fe–Ca (*r* = 0.997867; *P* < 0.05) in the gills and Fe–Zn (*r* = 0.997237; *P* < 0.05) in the skeleton were observed in B2 group. Also, positive correlation coefficients between Mg-P in the meat (*r* = 0.999855; *P* < 0.05) and in the skeleton (*r* = 0.995238; *P* < 0.05) were calculated in B2 group (Table 2).

**Table 2:** Effect of GOS supplementation on mineral correlation in tissues of common carp (*Cyprinus carpio* L.)

Tissues	Group	Minerals	Correlation coefficients values (r)			
			Ca	P	Mg	Zn
meat	C	P	− 0.848689			
		Mg	0.578071	− 0.059033		
		Zn	− 0.522277	− 0.007777	**− 0.997767**	
		Fe	0.971402	− 0.949999	0.367790	− 0.304856
	B1	P	0.225148			
		Mg	−0.039164	0.033006		
		Zn	0.053271	− 0.218170	− 0.211447	
		Fe	− 0.539374	0.398583	0.390499	0.037665
	B2	P	0.324344			
		Mg	0.308206	**0.999855**		
		Zn	0.526919	0.974871	0.970941	
		Fe	**− 0.999823**	− 0.342066	− 0.326032	− 0.542800
gills	C	P	0.451383			
		Mg	0.102977	**0.831992**		
		Zn	− 0.489005	0.075579	−0.082084	
		Fe	− 0.342147	0.261913	0.422110	0.477769
	B1	P	− 0.084785			
		Mg	0.585839	− 0.857180		
		Zn	0.759106	− 0.712984	0.972276	
		Fe	0.244280	− 0.986924	0.928984	0.816681
	B2	P	0.472731			
		Mg	0.826345	− 0.105625		
		Zn	0.197073	− 0.770763	0.714970	
		Fe	**0.997867**	0.529242	0.787824	0.132660
skeleton	C	P	0.630995			
		Mg	0.759933	0.275228		
		Zn	− 0.216576	− 0.503354	− 0.547748	
		Fe	0.673275	0.240347	**0.990319**	− 0.631448
	B1	P	0.531779			
		Mg	0.767650	0.380443		
		Zn	− 0.481985	− 0.396673	− 0.535817	
		Fe	− 0.788896	− 0.525375	− 0.398765	− 0.10209
	B2	P	− 0.826754			
		Mg	− 0.767979	**0.995238**		
		Zn	− 0.455535	0.877420	0.920004	
		Fe	− 0.388142	0.839356	0.888347	**0.997237**

Correlation coefficients marked in bold are statistically significant with *P* < 0.05; B1–1% Bi^2^tos®, B2–2% Bi^2^tos®

## Discussion

### Calcium Concentrations

Calcium (Ca) is one of the most abundant cations in the fish body and affects the structure of the skeletal system and maintaining a proper acid-base balance. Absorption of Ca from the gastrointestinal tract is controlled by hormones such as parathyroid hormones (PTH), calcitonin, and 1,25-dihydroxycholecalciferol [3]. As confirmed by research, muscle tissue is not the main site of Ca accumulation in fish as opposed to the fish scales, bones, and skin [3, 47]. Our results on Ca accumulation in various tissues of common carp were in agreement with Brucka-Jastrzębska et al. [48] and Łuczyńska et al. [49]. Numerous studies have confirmed that prebiotics such as oligofructose, inulin, galactooligosaccharides, resistant starches, and lactulose effectively stimulate Ca absorption [2, 50]. It has been hypothesized that short-chain fatty acids (SCFA), acetate, propionate, and butyrate, and other organic acids (e.g., lactate) produced by prebiotics lower the pH of light in the large intestine, which is associated with an increased amount of soluble Ca, especially in the caecum [33]. Studies have shown that the effect of the prebiotic on Ca absorption depends on the content of this mineral in the diet. Effect of GOS on Ca absorption was more effective when dietary Ca was higher than the recommended level [50, 51]. Our analyses showed no statistically significant differences in Ca content between fish fed 1 and 2% GOS compared control treatment (0% GOS) for each tissue. Therefore, further research is necessary to analyze the effect of different amounts of Ca in the diet on the effectiveness of the prebiotic activity on Ca absorption. Ortiz et al. [33] determined that content of Ca in the fillet of rainbow trout (*Oncorhynchus mykiss*) was not affected by prebiotic supplementation at inclusion level of 5 g kg^−1^ (FOS oligofructose BENEO P95; Beneo-Orafti Espanã SL, Barcelona, Spain). The lack of effect of the prebiotic on the absorption of Ca from the intestine may be due to the fact that the duration of the experiment was too short [50].

### Magnesium Concentration

Magnesium (Mg) is a cofactor of almost 300 enzymes, and thus participates in the transformation of carbohydrates, proteins, lipids, and nucleic acids. This metal plays an important role in the transmission of information between muscles and nerves, and it inhibits the process of blood clotting, owing to which it prevents the formation of clots. Mg, like Ca and phosphorus (P), is necessary for bone mineralization and it accumulates in the greatest amounts in bones [52]. Our results regarding the level of Mg accumulation in various tissues were in line with Brucka-Jastrzębska et al. [48], Brucka-Jastrzębska and Protasowicki [53], Brucka-Jastrzębska and Kawczuga [54], and Łuczyńska et al. [49]. The results of the present study showed that the highest level of this mineral was detected in the skeleton of fish fed 1% GOS (2.51 g kg^−1^ d.w.), and was significantly higher compared control treatment (0% GOS) (2.11 g kg^−1^ d.w.). However, 2% GOS supplementation did not cause a significant increase in Mg content. As Guerreiro et al. [55] confirmed, it is possible that fish gut bacteria community and digestive enzymatic activity had to adapt to the dietary modification. The results of studies on the effect of prebiotics on animal health are often contradictory, as fermentability of prebiotics may be affected by several factors, such as the type and dose of the prebiotic for example. The study of Biggs et al. [56] demonstrated that excessively high prebiotic dose may have a negative impact on the gastrointestinal system and may delay the growth of animals. This could be related to the inability of gut bacteria to ferment the high amount of prebiotic provided in the diet. The opposite hypothesis is that GOS, as a prebiotic with a low degree of polymerization (PD), at a dose of 1 and 2%, proved to be too weak in relation to the enzymes responsible for mineral metabolism. So, further research is needed to understand this mechanism. Our analyses confirmed no statistically significant differences in Mg content between treatment groups, both in the case of the meat and the gills. These results were in line with Ortiz et al. [33], who demonstrated that Mg content in the fillet of rainbow trout was not affected by prebiotic supplementation at inclusion level of 5 g kg^−1^. Factors that can reduce Mg absorption are fiber, phytates, P, Ca, or vitamin D.

### Phosphorus Concentration

Phosphorus (P) is a structural component of DNA, RNA, and phospholipids, involved in the photosynthesis and synthesis of organic compounds and responsible for the proper condition of teeth and bones. Fish can be a rich source of P; the concentration of which, depending on the species, can reach up to 200 mg per 100 g of meat. Phosphorus deficiency in the fish bodies can lead to excessive fat accumulation and poor skeletal mineralization and deformity [3]. It has also been shown that some phosphorus-containing compounds, such as phosphatidylinositol, play a very important role in preventing skeletal deformities [57]. Because the content of this mineral in the water is too low, and moreover, the efficiency of its absorption from feed is low, P should be supplemented with the feed [58]. Our studies have shown that as is the case of Ca, P concentration increased in the following order: meat < gills < skeleton (Figs. 1 and 2), but there were no statistically significant differences in P content between fish fed 1 and 2% GOS compared control treatment (0% GOS) for all tissues. However, noteworthy is the moderate increase in P of 2.33% (in the meat) recorded between C and B2 groups. Similarly, Ortiz et al. [33] demonstrated that content of P in the fillet of rainbow trout was not affected by FOS supplementation at inclusion level of 5 g kg^−1^.

In vertebrates, calcium forms a complex with phosphorus as hydroxyapatite, which is responsible for the structure and the mechanical strength of bones [59], and therefore, the Ca/P ratio is the most important indicator of good bone health because it prevents the reduction of bone mineral density [7]. The ratio of Ca to P in the whole body of several fish species ranges from 0.7 to 1.6 [7]. As numerous studies show, the value of this ratio should be 1:1 in consumed products, because the excess of calcium over phosphorus causes the formation of calcium triphosphate, which is not absorbed as this form of calcium triphosphate is not biologically available [59, 60]. Our analyses confirmed that Ca/P ratio in the skeleton was affected by GOS supplementation and its value increased from 1.91 (C group) to 2.25 (B1) and 2.22 (B2). Our results were similar to those obtained by Nwanna and Swartz [12] for common carp fed phytase.

### Zinc Concentration

Zinc (Zn) plays an important role in the proper functioning of an organism, especially of the immune system; it is a component of many metalloenzymes, regulates metabolism of carbohydrates, proteins, nucleic acids, and participates in insulin synthesis and in bone mineralization [60, 61]. The reduced rate of Zn uptake may be due to the presence of high amounts of calcium phosphate in feeds containing vegetable proteins, fiber, oxalates, phytates, and Fe [6]. Our results regarding Zn concentration in various tissues were in agreement with Bochenek et al. [62], Brucka-Jastrzębska et al. [48], Papagiannis et al. [63], and Jabeen et al. [64]. Prebiotic effect analyses revealed significantly higher amounts of Zn in the meat of fish from B1 and B2 groups in comparison with C group. Prebiotics like FOS and GOS promote growth of *Bifidobacterium* spp. that affect the synthesis of vitamin B6, which is responsible for better Zn absorption [65]. Since there is little data in the literature on this topic with regard to carp, further research is required. On the other hand, prebiotics affect the production of short-chain fatty acids, lowering the intestinal pH, which may contribute to better absorption of Zn. As confirmed by numerous studies, the gills play active and passive roles in exchanges between the body and its aquatic environment, and this tissue is the major storage site for Zn [3, 66, 67], which our research confirmed. However, our studies did not confirm a significantly positive effect of GOS supplementation on Zn concentration in the gills in B1 and B2 groups compared to C group, in contrast to the results obtained by Madreseh et al. [23] for rainbow trout lactulose-fed.

### Iron Concentration

Iron (Fe) is a cofactor of many enzymes and a component of blood and muscle chromoproteins. This metal supports the proper functioning of the nervous and immune systems. In addition, Fe is responsible for the detoxification of harmful substances in the liver and prevents anemia, as it is responsible for the production of red blood cells. Fe content of the muscles of fish is an important criterion for their suitability for consumption. Factors that inhibit Fe absorption from intestine are fiber, phytates, polyphenols, and tannins [3]. Fe deficiency causes anemia, but its excess leads to the formation of reactive oxygen species, resulting in cell and tissue damage. Therefore, Fe homeostasis must be strictly controlled to maintain balance [68]. Carp analyses showed that the tendency of Fe accumulation in various tissues was similar to those determined by Brucka-Jastrzębska et al. [48], Brucka-Jastrzębska and Protasowicki [53], and Tekin Özan and Aktan [69]. As our analyses indicated, GOS addition caused significantly higher level of Fe in the meat of fish fed 2% GOS in comparison with C and B1 groups. These results were in contradiction with Ortiz et al. [33]. Research confirms the beneficial effect of prebiotics on Fe absorption because fermentation of these substances by the natural intestinal microflora may lower pH and promote the reduction of Fe(III) to Fe(II), whose solubility is better. Moreover, prebiotics can stimulate the proliferation of epithelial cells to increase the absorption surface and stimulate the expression of proteins responsible for the transport of minerals in epithelial cells [24]. In our previous study with the same fish [36], a significantly accelerated development of the intestine, an increase in the height and width of the villi and an increase in their surface due to the GOS were found. As confirmed by research, propionate which is produced by intestinal fermentation of oligosaccharides may stimulate promoting 6-aminolevulinate synthesis (γ-keto carboxylic acid and precursor to the synthesis of porphyrins). Also, oligosaccharides may lead to a change in iron-binding proteins, e.g., mucin and enhance Fe absorption in the small intestine [70]. Carp analyses indicated that the tissues which contained the largest amounts of Fe were the gills. Fe is taken up by the gills in the form of free ions and chelates of this element [34]. The hypothesis is that in a highly alkaline and oxidizing environment, Fe uptake through the gill epithelium is limited. Our research supports this hypothesis as we have observed a significantly higher concentration of Fe in the gills of fish fed 1 and 2% GOS, which may be due to the prebiotic lowering environmental pH, but this mechanism requires further research.

### Minerals Interactions

Antagonist relationships occur when minerals have a similar electronic configuration and ion radius and compete for binding sites. Synergistic relationships occur when one element strengthens the role of another. In turn, the complex interrelationships between Fe, Zn, and Cu are more complex [3]. Understanding the mechanism of the correlation of one element with another can be used as an indicator of their co-location in biological tissues [71]. Studies of Wepener et al. [72] have shown that Cu (and possibly Fe) will have a greater tendency to accumulate in the gills than, for example, Zn or Ca. Our analyses confirmed positive correlation between Fe and Ca in the gills in B2 group which indicated that Ca had affinity for divalent metal transporter (DMT1) [71, 73]. In can be concluded that Fe and Ca may share uptake pathway via the DMT-1. However, it should be taken into account that the interaction between Fe and Ca depends on the form of calcium. Carbonates and phosphates, unlike calcium citrate, inhibit Fe absorption. Despite the fact that GOS supplementation did not affect the level of Ca accumulation in carp tissues, our analysis showed that 2% GOS addition caused a statistically significant increase in Fe in the gills. This could have had a significant impact on the positive correlation between these minerals in B2 group.

Positive correlations between Fe and Zn and between Mg and P in the skeleton in B2 group were calculated, despite the fact that these elements are considered antagonists to each other. Despite the fact that our studies confirmed the greatest accumulation capacity of Zn in the gills and Zn content in the skeleton was lower, this metal confirmed a synergistic interaction with Fe in the group of fish treated with 2% GOS supplementation. The interaction between Fe and Zn depends on the ratio of these metals to each other. The lack of published data on this topic requires further research on Zn metabolism, especially based on the analysis of vitamins and other minerals affecting Zn absorption.

Analyses confirmed positive correlation between Mg and P in the meat in B2 group. Although P and Mg are mainly involved in the bone mineralization process, their presence in the muscles is essential for the proper functioning of the organism. Both of these macroelements are responsible for conducting nerve impulses. Therefore, taking into account their proven antagonistic effect, the use of 2% GOS supplementation should be considered positive with regard to the correlation between these two elements. Due to the fact that there is no available published data about the effects of GOS on the relationship between minerals in carp tissues, this requires further analyses.

## Conclusions

The results from the current study showed that dietary GOS supplementation had a positive effect on absorption of some minerals in common carp tissues. The feed with 1 and 2% of GOS supplementation significantly enhanced the concentration of Zn in the meat and skeleton. Fe concentration was significantly higher in the B2 group compared to B1 and C in the meat and gills. One percent and 2% GOS supplementation significantly enhanced Fe content in the skeleton. Mg concentration was significantly higher in the skeleton of fish from B1 group compared to C group. A significant effect of 1 and 2% GOS supplementation on Ca/P ratio in the skeleton was also confirmed. Supplementation of feed with 2% GOS significantly influenced the positive correlations between Mg and P in the meat and skeleton, Fe–Ca correlation in gills, and Fe–Zn correlation in the skeleton. Based on the analysis of the mineral profile in different tissues, we can conclude that the prebiotic could be a potential dietary additive for farmed common carp, due to the significant increase in Fe and Zn, for example. The increased absorption of certain minerals may be caused due to the production of short-chain fatty acids, lowering the intestinal pH and regulating the factors responsible for the transport of divalent metals. Absorption of metals may be enhanced by vitamin synthesized by intestinal bacteria, which population is supported prebiotic. Another factor enhancing the minerals absorption may be an increase of the intestine development, an increase in the height and width of the villi, and an increase in their surface as a result of GOS supplementation, which was confirmed by our previous research on the same fish. Nevertheless, further research is needed in this topic to determine the detailed effects of GOS supplementation on minerals retention and on the interactions between minerals in different fish tissues.

## Data Availability

The datasets generated during and/or analyzed during the current study are available from the corresponding author on reasonable request.
